# Ecological migrants’ socio-spatial integration in Yinchuan City, China

**DOI:** 10.1371/journal.pone.0275853

**Published:** 2022-10-19

**Authors:** Zhao Ru, Long Dongping, Li Jing, Yang Meiling, Wen Xinyu

**Affiliations:** 1 School of Geography, South China Normal University, Guangzhou, China; 2 School of Geography and Remote Sensing, Center of GeoInformatics for Public Security, Guangzhou University, Guangzhou, China; 3 The Institute of Management, North Minzu University, Yinchuan, China; 4 School of Geography and Planning, Ningxia University, Yinchuan, China; Institute for Advanced Sustainability Studies, GERMANY

## Abstract

Promoting the social integration of ecological migrants and identifying the key constraints to their integration are significant issues for social governance and transformation. Most previous studies have not systematically analyzed the level of social integration of migrants from the perspective of spatial ternary dialectics or systematically analyzed physical, social, and spiritual spaces. Based on space production theory, this study used principal component analyses to evaluate the ternary spatial integration level of physical, social, and spiritual spaces of ecological migrants in a specific resettlement area in Yinchuan City, China, and the Tobit regression model was used to identify the key constraint factors. The results demonstrate that the overall socio-spatial integration levels of ecological migrants in Yinchuan City are lower than that of the overall migrant population nationwide, and the levels of their spatial adaptation and spatial practice integration lag behind that of spatial belonging and spatial identity integration. Meanwhile, length of residency, occupation type, monthly income, and household type have facilitating effects on the ecological migrants’ social integration, while gender, age, ethnicity, and education levels have insignificant effects. In particular, occupation type is a key factor in promoting social integration and improving employment among ecological migrants. It is concluded that upgrading production skills and raising employment for ecological migrants can foster sustainable social space production patterns, facilitate virtuous cycles, and eliminate inhibiting factors such as lagging spatial practices, regional cultural differences, and socio-spatial deprivation.

## Introduction

Urbanization is the movement of people and goods from rural areas to cities and involves transforming migrants’ production and lifestyles [1]. The main centers of the current gradual global urbanization are Asia and Africa, with China playing a central role [2]. Over time, the urbanization of parts of China has been completed as 227 million rural residents have moved into cities [3].

In 2014, the Chinese government has implemented New Urbanization Plan to develop people-centered urbanization and promote the active integration of migrants into cities [4]. However, the New Urbanization campaign led to several social problems. Owing to different migration purposes and different types of migration (labor migrants, entrepreneurial migrants, tourist migrants, etc.), the overall level of migrants’ social integration has been low [5–8]. For example, rural residents engaging in the informal economy in the cities also made them highly vulnerable, making it difficult for these migrants to obtain urban citizenship. The main cause of these problems is the discrepancy between the requirements of urban development and the possibilities for integration. Therefore, it is necessary to understand the social integration level and the driving factors of the different types of rural migrants.

At present, although researchers have not agreed on a definition of migrants’ social integration, there is consensus on some of its features. They agree that the goal of migrant social integration is to ensure that social groups can obtain the necessary opportunities and resources through which they will be able to participate fully in economic, social, and cultural life while enjoying equal social welfare benefits [9]. Previous research on the social integration of migrants has considered them a homogeneous group, which is common among many scholars. In other words, researchers have focused only on the social integration level and influencing factors of migrants at a macro level. As a result, migrants’ social integration is considered a homogeneous development process rather than a heterogeneous process. This assumption is contradicted by the findings of some scholars that labor migrants, entrepreneurs and tourist migrants have different social integration capacities [10]. This is due to the strong correlation between long-term planning and individual livelihood capital. Therefore, it is more necessary to assess the level of social integration and the driving factors of different types of migrants from the perspective of migration purposes to improve the overall social integration capacity of all migrants.

Meanwhile, another gap is the role of key factors in hindering social integration on different scales. There is evidence that immigrants experience low levels of social integration under the “rigid scale” of political migration. Ecological migration is a typical policy migration for sustainable ecosystem governance, and as part of the government initiatives to restore and recover severely degraded ecosystems, it typically relocates large numbers of people from traditional settlements with high ecological sensitivity to other areas with low ecological sensitivity [11]. Immigrants experience high levels of social integration under the “soft scale” of their voluntary migration for better development opportunities [12, 13]. Macro-level studies, however, tend to homogenize different levels of social integration capacity and the influencing factors of immigrants, and they fail to identify the differences between policy and voluntary immigrants or the driving factors behind their social integration level. Wang and Fan’s [14] study indicated that China’s migrants improved in terms of economic and cultural levels and the urban citizenship identity dilemma. However, Mao et al. [11] found that 72% of ecological migrants could not adapt to their new jobs in the Sanjiangyuan region of China, also further underscoring the differences between policy and voluntary immigrants.

To fill these gaps, this study will explore the degree of social integration and driving factors of ecological migration by collecting data from five ecological immigration communities in Yinchuan, China. More specifically, this study will evaluate the social integration levels of ecological migrants across different dimensions, which include social adaptation, social practice, social identity, and social belonging, and thus contribute to the research on migrants’ social integration from the following two aspects.

First, this study extends the existing literature by focusing on the subjective perceptions of migrants. For example, this study analyzes migrants’ self-identity concerning social integration through four dimensions: social adaptation, social practice, social identity, and social belonging. In addition, migrants are more likely to exhibit a higher level of social adaptation, while their levels of social practice reflect the ability to participate in urban social production and construction [15]. Also, social identity and social belonging are related to institutions and culture, such as social production patterns, governance, and cultural practices. For these reasons, we consider the social integration of ecological migrants to be a complex process. Consequently, this study is an analysis of the complexity of the social integration of ecological migrants and its limitations.

Second, we will select policy migration as the research object based on migration purposes. We do so to consider the heterogeneity of migration on a “soft scale” of intentions, rather than homogenizing the level of social integration of immigrants. Referring to Mao et al. [11] and Yang et al. [16], we divide rural migration into political “rigid” migration and voluntary “soft” migration. As the Chinese ecological immigration program is a typical “rigid scale,” we chose migrants who participated in the ecological migration program for this study. China’s ecological migration refers to the migration process. Migrants voluntarily relocated to other areas to settle and integrate into local production. These migrants live on nature reserves, severely damaged ecological environments, and ecologically fragile areas where human survival is difficult [17].

This paper is structured as follows: part 2 will describe the existing literature on migrants’ social integration; part 3 will explain the variables, data, and methods used in this study; and parts 4 and 5 will conduct calculations and analyze the findings.

## Literature review

The international research on migrant social integration is rooted in the macro theory of neoclassical economics, which emphasizes the role of the heterogeneity of immigrants’ skills in social integration. In other words, geographic differences in the supply and demand of labor and wage differences cause the migration of investors from capital-rich to capital-poor regions. High-skilled labor flows from capital-poor countries to capital-rich countries for high-skilled returns, while the parallel flow of managers and high-skilled labor is more active in local areas [18]. Consequently, immigration heterogeneity has been a focus of population research. In addition to micro factors, structuralism emphasizes the role of macro factors in the social integration of immigrants, which include politics, institutions, and cultural background.

Massey and España [19] found that external shocks (depression, war, or strict law enforcement) inhibited the integration process of international immigrants. Saksena and McMorrow [20] also demonstrated that history, power relations, and cultural differences could make the complete integration of political refugees impossible. These claims are supported by Kuiper and Greiner [21], who suggested that economic and market factors prevent the complete social integration of labor migrants. Peters et al. [22] analyzed the construction of a “memory network” in the social integration of more than 70 immigrants from Poland, the Netherlands, and Germany. They found that history and local and social relationships are the foundation for entertainment and a sense of belonging. In the 1970s, the theory of space production began to be applied to study cities, political power, and daily life [23]. This theory is highlighted in urban studies as people abandon their rural capital and move to cities (forced or voluntary), living on the urban periphery and working in low-paid jobs. This leads to a lack of spatial belonging and identity of groups, resulting in territorial differentiation of social groups and the formation of distorted social relations and mutually exclusive spatial perceptions that hinder the process of socio-spatial integration in cities [23].

Scholars have used the above theories to study the social integration of immigrants from rural areas in China, and the initial empirical research results demonstrate that the “soft scale factor” of willingness significantly impacts the social integration of immigrants [7, 24, 25]. Chinese migrants’ social integration factors can be divided into three categories. While micro-scale migration and social integration characteristics are significant (e.g., age, sex, and education level), mesoscale social relations (e.g., the closer they are to the cultural customs and kinship of the destination country, the faster and higher the social integration of immigrants), national macroeconomic policies, and geographical factors are also important (e.g., the hukou system, which significantly influences migrants’ willingness to settle and the speed of social integration).

However, in terms of the influence of national policies, existing studies have mainly focused on urbanization, a national policy with guiding properties, and less on ecological immigration, a national policy with rigid properties. For example, Tao et al. [26] studied the housing satisfaction of rural migrants in China and found that formal housing situation and intention to settle in the city were positively correlated. Liu et al. [25] argued that the urbanization process of immigrants living in traditional housing implies a higher level of social integration in terms of willingness to settle. Yang et al. [16] found that community support, community functioning, age, and marital status influence the social integration of migrant workers in development enclaves; occupational skills, marital status, and age influence the social integration of migrant workers in mixed and stable enclaves; and community support and length of residence influence the social integration of migrant workers in industrial enclaves. Their study demonstrates that urbanization programs promote more research results on the social integration of farm-named workers. By contrast, only Mao et al. [11] and Tai et al. [17] have analyzed the adaptation of ecological immigrants to new settlements under ecological migration programs in China.

Tai et al. [17] demonstrated that ecological migrants’ original organizational structure and social networks were weakened, as well as their cultural characteristics, customs, and reciprocity network were damaged, which reduced migrants’ sense of social identity and recognition of community norms. Meanwhile, ecological migrants are limited by their own production experience, literacy, and technical operation levels, which weren’t to build a solid space production model and social organization, and formed an imbalanced development model of material, social, and spiritual space. Mao et al. [11] and Jeworrek et al. [27] found that most ecological migrants had to rely on the living allowances provided by the government and that 72% were unsuitable for the new job requirements in ecological resettlement areas. Most of the migrants worked in the informal economy in exchange for the capital needed for education, medical care, and living expenses. This intensifies the pressure on infrastructure and public service facilities in the relocation area. Hedlund [28] argued that this might lead to migrants settling in small towns having busy day-to-day lives with multiple everyday activities and practices outside the local area, and their lack of interaction with city dwellers in production and daily life, resulting in social isolation and spatial breaks in their social practices. Migrants lost the opportunity to accumulate experience for space production practice and weakened spatial identity, and sense of belonging, thus hindering their socio-spatial integration.

As Romoli et al. [29] illustrated, ecological migration involves the optimal allocation of population and resources and reflects the conflict and integration of social and cultural spaces. This is evidenced by the fact that residents who have relocated face problems such as weakened spatial adaptability [17], lack of spatial identity and sense of belonging [30], imbalance of space production relations, and spatial perception shock (e.g., social exclusion) [31–33]. Mao et al. [11] found that ecological migrants move to resettlement areas to work and live. However, due to their different spatial allocation, adaptation, and practices relevant to their knowledge and culture, spatial conflicts and social disadvantages may arise. This, in turn, inhibits their space production levels and organizational efficiency, resulting in inferior positions in production and life and hindering their socio-spatial integration. Therefore, ecological migrants adapting and integrating into society is an urgent research issue in urban governance. Based on the Western theory of “space production,” this study aims to understand the processes and levels of social integration of Chinese ecological migrants. In particular, this study contributes to the research in the international literature on the role of migrant heterogeneity in social integration by identifying the key factors that constrain the social integration of ecological migrants.

## Data and methods

### Framework

**Fig 1:**
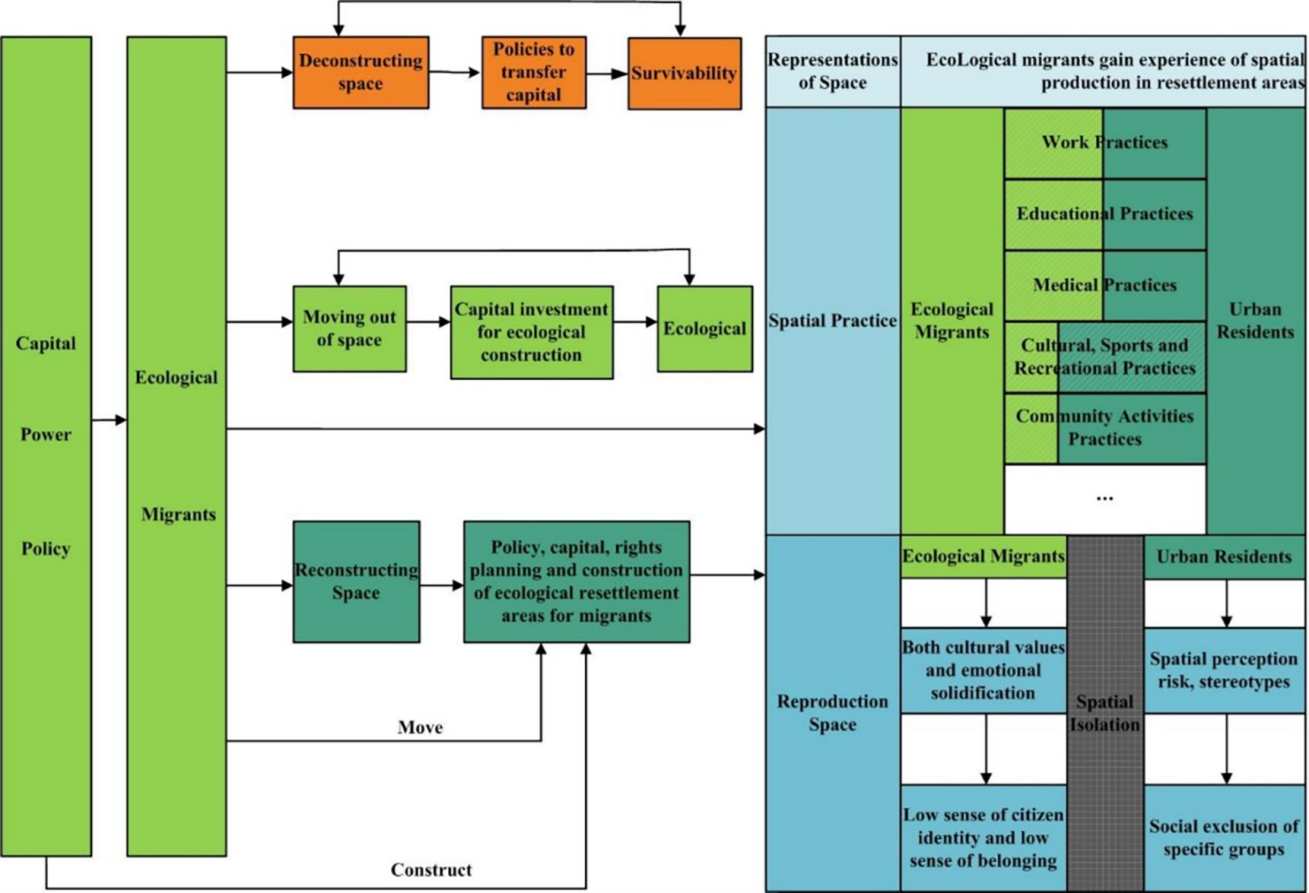
Social space production structure of ecological migrants.

Lefebvre’s [34] theory of space production includes three systems: *representations of space*, *spatial practice*, and *reproduction space*, which were then developed by Soja, to create the space production paradigm [35]. The triadic dialectical analysis paradigm corresponds to *material space*, *social space*, and *spiritual space* types. Generally, representations of space reflect the material connotation of the mainstream social order in the physical space. However, the understanding and environmental perception of the material connotation of the same physical space varies between different groups. Spatial practice reflects the process of shaping real space through power, capital, and productivity and constructs the social network space of groups through daily practices and production activities. Reproductive space reflects the privacy of social life and production while challenging and critiquing common socio-spatial practice and its spatiality by taking the perceived value of space constructed in material space and social space as a reference [36]. The triadic space production process reveals the interaction between social production and spatial configuration, and social production and life are thus considered dynamically changing space production processes [35]. Hence, this paper argues that the social integration of ecological migrants can also be regarded as an interactive process of social space production. The interaction of social space production of ecological migrants reflects the whole process of “deconstructing space-moving out of space-reconstructing space” [37] in human society (Fig 1), which is driven by material and institutional factors such as government capital, power, and policies.

The Chinese government introduced social capital in 1996 to aid the construction of supporting facilities in ecological resettlement areas. This ensures that people can “move out of the area” [38], and they are “ready to live.” The key to being “ready to live” is to build a solid space production model and social organization. However, compared to urban residents who have formed a balanced development model of material, social, and spiritual space, ecological migrants, in the process of acquiring living and production materials in the relocation area, are limited by their own production experience, literacy levels, and technical operation levels. They mostly engage in manual labor in exchange for the capital required for education, medical care, and living expenses, resulting in their social space production interaction processes as mostly concentrated in the fields of work, education, and medical care. This intensifies the pressure on infrastructure and public service facilities in the relocation area [38], which may lead to the rejection of ecological migrants by the original urban residents, resulting in the phenomenon of social isolation and spatial break in their social practices. This, in turn, reduces the space production experience, spatial identity, and sense of belonging of ecological migrants, thus hindering their socio-spatial integration [39].

This study focuses on the level of social integration in the triadic space of ecological migrant groups. It identifies the key factors that constrain the improvement of the space and uses the reproduction and development levels of materials, organizational relations, and social network spaces of the migrants to promote their social integration.

### Variables

Existing studies assessing the degree of social integration of ecological migrants refer to the willingness to settle, social wellbeing [31–33, 40], and cultural adaptation [41], which overlap and differ slightly. Based on the central thesis that social integration of ecological migrants is an interaction of subjective and objective, internal and external factors, this study argues that the activity and interaction process of spatial representations, spatial practices, and representational spaces of ecological migrants at the resettlement site corresponds to the interaction process of material space, social space, and spiritual space in spatial types. Specifically, the more ecological migrants adapt to the environmental conditions and lifestyles of the areas to which they move, the more actively they participate in community cultural practices, express their wishes, and practice mutual assistance with urban residents, the stronger their sense of spatial identity and belonging becomes. When social production and organizational relations become more harmonious, they promote the development process of social integration [42]. The degree of social integration depends on the combined result of representations of space, spatial practice, and the spaces of representation. The triadic dialectic of space production and its correspondence in spatial types formed the basis for measuring the level of social integration of ecological migrants in this study. On this basis, the index system of the study was constructed by drawing on research findings on the social integration of mobile populations. For example, Lin et al. [41] selected indicators of neighborhood interaction composition, willingness to integrate, and attachment to the city to analyze the level of social integration of migrants across Chinese neighborhoods. They found that the number of immigrants living in commercial housing was higher than those living in factory dormitories and old neighborhoods. Huang et al. [3] selected indicators of community service, frequency of neighborhood interaction, and channels for seeking outside help to study the settlement intentions of Migrant workers in large cities in China. Liu et al. [7] argued that migrants’ social practice activities and relationships significantly affected their settlement and integration into the city. Chen et al. [43] studied the perceived opportunities for social integration of immigrants in China and found that the degree of familiarity and mutual assistance from neighbors was positively related to the immigrants’ perceived social status. Wu et al. [44] examined the variables of willingness to participate in community activities, sense of belonging to the community, degree of local attachment, and willingness to stay to investigate the degree of social integration of urban migrants in Guangzhou. They found heterogeneity in immigrants’ local connectedness and institutional barriers to social participation.

Accordingly, three indicators were selected in this study to characterize the representation of spatial integration levels in ecological migrant communities for physical spatial adaptive capacity: community living environment and conditions, community lifestyle, and neighborhood interpersonal relations. Two indicators were selected to characterize the degree of integration of ecological migrants’ spatial practices at the socio-spatial level: the frequency of discussion of community issues and activities to which ecological migrants were invited and the willingness to participate in community activities. At the spiritual space level, the degree of integration of ecological migrants was assessed together with the degree of spatial identification and spatial belonging. The degree of identification n with community leaders, satisfaction with community services, and satisfaction with living status in the community were selected to characterize their degree of spatial identification. Community affinity, community attachment, and citizen identity characterized the degree of spatial belonging. In this work, three subsystems with four primary and eleven secondary indicators were created to multidimensionally assess the degree of socio-spatial integration of ecological migrants (Table 1).

**Table 1:** Evaluation index system of the social space integration level of ecological migrants.

Subsystem	Primary indicators	Secondary indicators	Type and assignment	Attribute
Representations of space	Spatial Adaptation (A)	Community living environment and condition adaptability (A1)	Very adaptable = 1, adaptable = 2, not too adaptable = 3, not adaptable = 4, especially not adaptable = 5	+
		Community lifestyle adaptation (A2)		+
		Neighborhood interpersonal relationship adaptability (A3)		+
Spatial Practice	Spatial Practice (B)	Frequency of discussions invited to participate in community affairs and activities (B1)	Always = 1, Often = 2, Sometimes = 3, Occasionally = 4, None = 5	+
		Willingness to participate in community activities (B2)	Very willing = 1, willing = 2, more willing = 3, less willing = 4, not willing = 5	+
representational spaces	Spatial identity(C)	Agreement with the level of community managers (C1)	Very satisfied = 1, satisfied = 2, more satisfied = 3, less satisfied = 4, dissatisfied = 5	+
		Satisfaction with community services (C2)		+
		Satisfaction with the state of community life (C3)		+
	Spatial Belonging(D)	Sense of community closeness (D1)	Very strong = 1, strong = 2, relatively strong = 3, not too strong = 4, no feeling = 5	+
		Sense of community attachment (D2)		+
		Citizenship identity (D3)		+

### Description of area

Yinchuan, the capital of the Ningxia Hui Autonomous Region, lies in the Ningxia Plain in western China. The Ordos Plateau lies to the east, the Helan Mountains to the west and the Yellow River flows through the city. In 1983, Ningxia conducted six large-scale ecological resettlements and resettled 1,323,600 migrants, a typical area for ecological resettlement [45]. For this study, an ecological resettlement community in Yinchuan, Ningxia, was selected as a case study. Yinchuan City is the core city of the Yellow City Cluster, which has economic agglomeration and cultural inclusiveness as well as a strong gravitational force for population migration, not only settling ecological migrants in the region but also attracting migrants from Gansu, Shaanxi, Anhui, and Zhejiang. For the selection of communities, five typical communities in which the number of ecological migrants accounted for 80% of the total population of the communities were selected for this study: Brick Drainage New Village, Taqiao Home, Youai Home, Shangqian Kangju, Zhuanqu Xincun, and Kangyuan Yaju.

### Survey and data

The researchers specifically referred to the method of surveying community participation through random sampling and the Kish sample of urban residents [44]. Urban residents were excluded according to the sampling requirements. The first question of the questionnaire was, “Are you an ecological migrant”? Adult ecological migrants were selected for the surveys based on the criteria that they moved to Yinchuan City after participating in the ecological migration project, rather than moving spontaneously or participating in other engineering projects, such as the land transfer project. The data collected were used to build a picture of the social integration of ecological migrants.

The research team adopted a survey as the research tool, which was distributed to 349 ecological migrants randomly selected via the stratified random sampling method [44]. For each ecological migrant, an interview of about 30–40 minutes was conducted to obtain data on the social integration of the ecological migrants. The questionnaire survey consisted of two parts: demographic information and Likert scale questions. Demographic information included gender, age, education level, and type of household registration (49.2% males, 50.8% females, age range 46 to 60 years, average education level was junior high school, and the average length of residence was 3.996 years). The survey, with a five-point Likert scale (1 for “very poor” to 5 for “very good”), included 11 questions on the four dimensions of spatial adaptation, spatial practice, spatial identity, and spatial belonging. A total of 349 questionnaires were distributed. They were all returned, but only 331 were valid, an effective rate of 94.84% (Table 2).

**Table 2:** Survey questionnaire distribution and validity.

Survey Communities	Valid questionnaires (copies)	Proportion (%)
Zhuanqu Xincun	80	24.17%
Taqiao Home	80	24.17%
Youai Home	51	15.41%
Shangqian Kangju	67	20.24%
Kangyuan Yaju	53	16.01%
Total	331	—

### Method specification

First, we used a principal component analysis to measure the social integration level of ecological migrants. Second, we used the Tobit regression model to identify the key factors affecting their level of social integration.

#### Principal component analysis

The principal component analysis method has significant advantages when evaluating multiple complex variables, as it reveals the internal structure of the variable using multiple principal components while retaining the most important information of the original variable. Two measurement steps were carried out. First, the extreme difference standardization method was used to eliminate the differences in the sample data. Second, SPSS software (version 26.0) was used to analyze the standardized sample data. These were scaled down to obtain a value of 887.543 for Bartlett’s sphericity test and a value of 0.791 for KMO sample fitness, which were suitable for a factor analysis based on Kaiser’s standard. Third, the correlation coefficient matrix Q and eigenvector α were determined to obtain the sample indicators’ variance contribution rate and the cumulative variance contribution rate. Fourth, the four components with eigenvalues greater than 1 were extracted with a cumulative variance contribution rate of 64.969%. Consequently, the four principal components reflect the main information of the sample variables. Fifth, the principal component scores were based on the principal component eigenvalues, standardized values, and rotated principal component loadings. Sixth, the degree of social integration was determined by adding and weighting the sum with the values of the principal components using the variance contribution of the four principal components as weights with the following formula [46]:

$$F_{p}=a_{1i}\times Z_{x1}+a_{2i}\times Z_{x2}+\cdots +a_{pi}\times Z_{xp}$$ (1)

where *a*_1i_,*a*_2*i*_,⋯,*a*_*pi*_(*i* = 1,⋯,*m*) are the eigenvectors corresponding to the eigenvalues of the covariance matrix of *X*. *Z*_*x*1_,*Z*_*x*2_,⋯,*Z*_*xp*_ are the normalized values of the original variables.

$$R_{ai}=\gamma_{i}a_{i}$$ (2)

where R is the correlation coefficient matrix, γ_i_ and a_i_ are the corresponding eigenvalues and eigenvectors, respectively, and γ_1_≥γ_2_,⋯,γ_*p*_≥0.

$$S_{ij}=\sqrt{\alpha_{j}}F_{ij}$$ (3)

$$T=\sum_{j-1}^{k}\left(\alpha_{j}S_{ij}\right)$$ (4)

where *F*_*ij*_ is the j_th_ unrotated factor score of the i_th_ sample, *S*_*ij*_ is the j_th_ principal component score of the i_th_ sample, *T* is the level of social integration of ecological migrants in Yinchuan, and α_*j*_ is the variance contribution rate of the j_th_ factor.

#### Tobit regression model

Since the T values range from 0 to 1, the traditional least squares regression is not the appropriate method to assess the determinants of ecological immigrants’ level of social integration [47, 48]. Therefore, the Tobit regression model is more suitable for measuring the determinants. Existing studies on city integration also apply the Tobit model [49, 50]. The variables were ascertained using the previous literature and the reality of the situation. The following model was then used to conduct the analysis:

$$T=\theta_{1}XB+\theta_{2}TIME+\theta_{3}AGE+\theta_{4}NAT+\theta_{5}PRO+\theta_{6}EDU+\theta_{7}MON+\theta_{8}REG+C+\tau$$ (5)

where *T* is the level of social integration; *XB*,*TIME*,*AGE*,*NAT*,*EDU*,*PRO*,*MON*, and *REG* are gender, time, age, ethnicity, education level, occupation type, monthly income level, and household type, respectively; θ_1_ to θ_8_ are regression coefficients; *C* is the intercept term; *τ* is the random disturbance term.

## Results

### Integration of immigration

Table 3 illustrates the results of the factor analysis. There are four dimensions of social integration of ecological migrants in Yinchuan, and the initial eigenvalues of the principal components are 3.563, 1.399, 1.157, and 1.027, respectively. Due to the large differences between the initial eigenvalues, the maximum variance rotation method was applied in this study to rotate the factor loading matrix and obtain the rotated component matrix to improve the interpretation of the factor naming (Table 3).

**Table 3:** Composition matrix after rotation.

Factor	Raw variables	Component 1	Component 2	Component 3	Component 4	Degree of commonness
A1	Community living environment and condition adaptation	0.772	0.107	0.129	-0.033	0.626
A2	Community lifestyle adaptation	0.798	0.217	0.068	0.029	0.689
A3	Neighborhood interpersonal adaptability	0.564	0.508	-0.179	0.113	0.621
B1	Frequency of discussion of invited participation in community affairs and activities	-0.063	0.007	0.662	-0.112	0.455
B2	Willingness to participate in community activities	0.06	0.734	0.228	0.120	0.609
C1	Satisfaction with community management and services	0.697	-0.164	0.252	0.009	0.577
C2	Neighborhood rapport	0.058	0.631	0.044	-0.185	0.730
C3	Community living state satisfaction	0.387	0.118	0.700	0.060	0.656
D1	Sense of community closeness	0.134	0.537	0.531	0.211	0.633
D2	Sense of community attachment	0.363	0.339	0.587	0.186	0.626
D3	Citizenship identity	0.005	0.002	0.008	0.961	0.925

As evident from Tables 3 and 4, Principal Component 1 has the strongest explanatory power for the information of the original variables. Its eigenvalue is 3.563, much larger than the eigenvalues of the other principal components, with a variance contribution of 21.344%. The most explanatory power indicators in principal component 1 were the spatial adaptation indicators (A1, A2) at 0.772 and 0.798, respectively. The variance contribution of principal component 2 is 18.093%, with the spatial practice indicator having the largest coefficient (B2) of 0.734. Therefore, principal components 1 and 2 can explain the characteristics of the spatial representation and spatial practice dimensions, respectively. The variance contribution of principal component 3 was 15.683%, with the coefficient of the spatial identity indicator being the largest at 0.700 (C3), and the variance contribution of principal component 4 was 9.849%, with the coefficient of the spatial attribution indicator being the largest at 0.961 (D3). These two indicators can explain the characteristics of the reproduced spatial dimension. The rotated principal component matrix contained negative values, and a data translation was used to eliminate them.

**Table 4:** Extraction results of main factors of social integration of ecological migrants in Yinchuan.

Dimension	Principal components	Name	Original features values			Sum of squared rotated loadings			Structural dimensional component score
			Total	Variance	Cumulative	Total	Variance	Cumulative	
Representations of space	S1	Spatial adaptation	3.563	32.389	32.389	2.348	21.344	21.344	35.028
Spatial practice	S2	Spatial practice	1.399	12.718	45.108	1.990	18.093	39.437	36.683
Representational spaces	S3	Spatial identity	1.157	10.521	55.629	1.725	15.683	55.12	48.64
Level of social integration	S4	Spatial belonging	1.027	9.339	64.969	1.083	9.849	64.969	40.516
oveerall			-	-	-	-	-	-	38.591

Among the four principal components, spatial adaptation had the highest variance contribution at 21.344%, followed by spatial practice, spatial identity, and spatial attribution factors, which were named S1, S2, S3, and S4, respectively, based on the variance contribution and eigenvalues (Table 4).

The social integration level of ecological migrants in Yinchuan and the scores of each principal component were obtained using a weighted summation and then converted to values between 1 and 100 (Table 4) using the variance contribution values of the four principal components. Overall, the degree of social integration of ecological migrants in Yinchuan was 38.591. In each dimension, the degree of integration decreases successively from spatial identity, spatial belonging, spatial practice, and spatial adaptation, while the gap between spatial practice and spatial adaptation and that between spatial belonging and spatial identity become wider. The comparison of different integration dimensions demonstrates that ecological migrants in Yinchuan have a relatively high degree of integration in spatial identity and belonging dimensions and a low degree of integration in spatial adaptation and practice dimensions. Although scholars both within and outside of China emphasize that integrating practices and adaptive behaviors are prerequisites for cultural and psychological integration [51], this study finds that the spatial adaptation and spatial practice integration of ecological migrants in Yinchuan lag behind cultural and psychological integration. Furthermore, it establishes that social integration is multidimensional and multilevel. With the compression of space, time, and diversification of information channels, the receptiveness, and tolerance of residents’ cultural customs generally improve, accelerating their cultural and psychological integration.

### Influence factors

#### Variables

The main classical theories that explain the factors influencing residents’ problems with social integration are social integration theory [52], world systems theory, and institutional theory [18]. Synthesizing the current empirical research results of scholars in the field, this study draws on Liu et al.’s [25] research ideas on the factors influencing migrants’ socioeconomic status and subjective wellbeing. These are explained from two perspectives: internal factors, such as demographic characteristics, and external factors, such as economic and social factors. As far as internal factors are concerned, the younger the age group, the better the ability to learn and the faster the acceptance of new things, and thus, the faster the social integration. Migration decisions are household-based and are influenced by income and livelihood patterns [53]. In general, older ecological migrants have higher levels of education and pursue better-paid occupations. They also tend to actively integrate into their new communities so that their social integration is faster and higher.

Regarding external factors, the level of urban economic development is closely related to the speed of social integration. Based on internal and external factors, a total of eight independent variables were selected: gender, length of residence, age, ethnicity, education level, type of household registration, type of occupation, and monthly income. The names, types, means, and standard deviations of the variables are listed in Table 5.

**Table 5:** Variable selection of influencing factors of social integration of ecological migrants in Yinchuan.

Independent Variables		Variable type and assignment	Mean	Standard deviation
Are you an ecological migrant?	Have you moved to the resettlement area because you participated in the ecological migration relocation project?	No (0)	-	-
		Yes (1)		
Gender	Your gender	Male = 1, Female = 2	1.508	0.500
Length of residence	Length of time you have settled in Yinchuan City since you moved there	Time you moved to the resettlement area	3.996	3.817
Age	Your age	Under 18 years old = 1, 19–30 years old = 2, 31–45 years old = 3, 46–60 years old = 4, over 60 years old = 5	3.538	1.317
Ethnicity	Your ethnicity	Han = 1, Hui = 2, Other = 3	1.785	0.418
Type of occupation	Your kind of work	unemployed persons = 1, military personnel = 2, informal economy workers = 3, operators of production and transportation equipment = 4, production personnel in agriculture, forestry, animal husbandry, fishery, and water conservancy = 5, commercial and service personnel = 6, clerical and related personnel = 7, Professional and technical personnel = 8	5.511	2.584
Education level	Your level of education	Below elementary school = 1, junior high school = 2, high school or junior college = 3, college = 4, undergraduate = 5, graduate and above = 6	2.118	1.187
Monthly income level	Your monthly income	Less than $1000 = 1, $1000–2000 = 2, 2001–4000 = 3,4001–6000 = 4,6001+ = 5	1.985	1.106
Household registration type	Your household type	non-agricultural households = 1, Agricultural households = 2	1.574	0.494

#### Estimation results

The Tobit model was constructed using the Stata 17.0 platform to identify the key factors influencing the social integration level of ecological migrants. Table 6 illustrates that gender, age, ethnicity, and education level had no significant influence on social integration. However, factors such as length of residence, occupation type, monthly income levels, and household registration type significantly impacted the confidence levels of 10%, 5%, and 1%, respectively. This suggests that these are key factors in the level of social integration of ecological migrants in Yinchuan.

**Table 6:** Regression results of factors influencing the social space integration of ecological immigration.

Variables	Overall	Zhuanqu Xincun	Taqiao Home	Youai Home	Shangqian Kangju	Kangyuan Yaju
	(t statistic)	(t statistic)	(t statistic)	(t statistic)	(t statistic)	(t statistic)
Gender	-0.010	-0.008	0.036	-0.011	-0.012	-0.061
	(-0.570)	(-0.290)	(1.090)	(-0.220)	(-0.250)	(-1.620)
Length of residence	0.111**	-0.509	-0.258	-0.173***	-0.091	0.207
	(2.360)	(-1.640)	(-0.770)	(-2.920)	(-0.840)	(0.640)
Age	0.005	-0.034	0.038	0.040	0.148	-0.016
	(-0.160)	(-0.500)	(0.650)	(0.820)	(1.650)	(-0.180)
Ethnicity	-1.300	-0.024	-0.119	0.021	0.206	-0.143**
	(-1.270)	(-0.250)	(-1.270)	(0.340)	(1.580)	(-1.920)
Occupation type	0.097*	0.173*	0.052	0.157*	0.278**	0.113
	(1.830)	(1.910)	(0.570)	(1.860)	(2.220)	(0.810)
Education levels	0.042	-0.016	0.152***	0.112**	-0.157	0.184***
	(1.200)	(-0.280)	(2.840)	(2.150)	(-1.600)	(3.080)
Monthly income levels	0.086*	-0.071	0.125**	0.045	-0.198	0.314***
	(1.930)	(-0.820)	(2.110)	(0.510)	(-1.200)	(2.930)
Household registration type	-0.071***	-0.018	-0.064	-0.077	-0.061	-0.164***
	(-3.580)	(-0.530)	(-1.630)	(-1.590)	(-0.950)	(-3.900)

Note

***^,^ ***, and * indicate significance at the 1%, 5%, and 10% levels, respectively

The length of residence had a significant positive effect on social integration and passed the p-test at the 5% significance level. The overall level of social integration of ecological migrants in Yinchuan is low, and their average length of stay is about four years, with a large standard deviation of 3.817, indicating that the differences in the length of stay of ecological migrants and their length of stay in Yinchuan are large. Of them, 43% had been living in Yinchuan for less than or equal to two years, 35% for three to four years, and 22% for five to nineteen years. Field interviews revealed that most ecological migrants had lived in the region for a relatively short time and were still in the learning and adjustment phase of production activities and lifestyles. The increasing population flow, combined with the fact that ecological migrants move to ecological migrant settlements and have not yet established strong emotional ties and productive relationships, means that their perception of risk in the community increases in parallel [54], which can slow down the overall process of social integration.

The occupation type positively impacts improving the social integration level of ecological migrants in Yinchuan. The occupation type has the second greatest effect on social integration. The type of occupation is an important indicator of social status. Professionals, technicians, commercial, and service workers use their professional knowledge and capital to obtain more means of production, improve the quality of life of their families, and access better education and service resources, which promotes their social integration. The reality of the situation has found that the ecological migrants in Yinchuan trying to obtain work can be classified as unemployed, informal economy workers, and production, professional and technical personnel and transportation equipment workers; they account for 6.3%, 45.3%, 6.6%and 4.8%, respectively. It indicating that ecological migrants moving into Yinchuan are mostly engaged in productive activities and obtained means of production, which promoted social integration. However, they may live on the city’s periphery, resulting in a “spatial break” between the social practices of ecological migrants and urban residents. There are few opportunities for access to quality educational resources and service facilities, and their quality of life improves only slowly, leading to “social isolation” between ecological migrants and city dwellers. This, in turn, has a restraining role in improving occupational type.

One of the main reasons for ecological migrants whose are informal economy workers in Yinchuan is that many come from the Liupan Mountains in southern Ningxia, where most residents are farmers. The path-dependent effect of their mode of production and spatial differences cause their spatial practices to lag behind. Therefore, the introduction of beneficial external variables, such as government funding and guidance, can provide platforms and pathways for transforming their mode of production, changing their path dependency, improving their employment and structure, and providing direction for social adjustment and integration.

The regression results demonstrate that the social integration of ecological migrants in Yinchuan City developed with the increase in income level and passed the p-test at a 10% significance level. The reason could be that the monthly income level indicates, to some extent, the socioeconomic and productivity level of the group, and the higher the monthly income level, the better the socioeconomic status and the stronger the social productivity. This result is similar to the findings for Swedish immigration flows [55]. The survey research demonstrates that the monthly income of 47.4% of the ecological migrants is below RMB 1,000; 19.6% are between RMB 1,000 and 2,000; 23.3% are between RMB 2,001 and 4,000; 7.9% are between RMB 4,001 and 6,000; and 2.4% are above RMB 6,000. The average monthly income level is 1,982, which corresponds to a monthly income level of about RMB 1,000–2,000, which is lower than Yinchuan residents’ average monthly income level (RMB 2,965.5) and the national average monthly income level of Chinese residents (RMB 2,657.9). These income levels indicate that ecological migrants accumulate less livelihood capital. Most of them rent their homes, while their housing, education, and medical care costs are high, exacerbating their capital vulnerability. This factor may even cause them to return to their original homelands.

The type of household registration significantly impacts the process and level of social integration of migrants in Yinchuan, passing the p-test at the 1% significance level. This indicates that non-agricultural household registration has a significant effect on social integration. With 42.6% non-agricultural household registration and 57.4% agricultural household registration, the institutional attributes of social integration for these migrants are clear, as non-agricultural migrants display better social integration. They have more livelihoods, and they can receive more external information, more open production space, and make stronger demands for a better-developed environment. These factors enhance their level of social integration and accelerate their integration process. This is consistent with Lin et al.’s [41] study on the social integration of migrants in China. However, most ecological migrants in Yinchuan are agricultural households and are slow to perform in social integration. It currently shows a negative impact on the level of social integration.

Additionally, Table 6 illustrates the results of the regression analysis of ecological migrants of five communities in Yinchuan. Length of residence, occupation type, monthly income levels, and household registration type produced almost identical impacts on social integration for each community overall. While significantly negative relations were found in the attention to age, ethnicity, and social integration for partial migrants, positive relations were noticed between education level and social integration for some ecological migrants. Specifically, the social integration of ecological migrants in Zhuanqu Xincun increased with occupation type. The social integration of ecological migrants in Taqiao Home improved with monthly income and education levels. Social integration of ecological migrants in Youai Home improved with occupation type and education levels but was slow for the length of residence. Social integration of ecological migrants in Shangqian Kangju improved with occupation type and education levels, but significantly negative relations were noted between age and social integration. Social integration of ecological migrants in Kangyuan Yaju improved with education levels and monthly income levels, while it was slow for ethnic minorities and those with agriculturally registered permanent residence.

## Conclusions and discussion

This study presents the results of a survey of 331 ecological migrants in five typical communities in Yinchuan from the perspective of the triadic interaction process of social integration. The degree of their social integration was measured, and the influencing factors were identified. The results confirm the applicability and scientific validity of the space production theory for studying ecological migrants in China.

This study demonstrated that the overall social integration of ecological migrants in typical communities in Yinchuan is low, and the levels of spatial adaptation and practice integration lag behind spatial belonging and identity integration levels. At the same time, this study found that the length of residence, the occupation type, the amount of monthly income, and the type of residence (agricultural or non-agricultural) affect the level of social integration differently. The length of residence, occupation type, and level of monthly income had a positive impact on the social integration of migrants, along with levels of regional economic development and production skills development. However, migrants’ social integration into agriculturally registered permanent residences is slow, and the level is low. Overall, an insignificant relation between gender, age, ethnicity, education, and social integration for ecological migrants was noted.

This study confirms that the length of residence had the greatest impact effect on the social integration of ecological migrants in Yinchuan. The average time of social integration for most ecological migrants is 3.996 years. Their social space production practices were low-skilled, and they did not participate in leisure activities. Urban residents living in the city tend to engage in skilled and productive activities and develop a work-life balance that includes leisure activities. This balance allows them to experience a “spatial break” in their social practices. When there is no spatial break, the social integration of ecological migrants is hindered. The key to promoting their social integration, therefore, lies in improving their production skills and cultivating sustainable production and living patterns to promote a positive cycle of social space production.

This study also identifies the level of social integration of ecological migration and its influencing factors under the “rigid scale” of policy. This research result improves the level of integration of material space, practical space, and social space of policy migration and solves the dilemma of the urban identity of immigrants. It also enriches the research content of urban social integration and explores the guiding nature of space production theory in urban research. Nevertheless, this study has several limitations. The types of migrants are diverse, and in this study, only ecological migrants are considered as a specific migrant group. In addition, the small sample size used in this study may make it difficult to comprehensively capture the complex spatial organization and productive relationships of all migrants moving to the city. Future studies should, therefore, use larger sample sizes and different types of migrants to conduct deeper analyses, explore migrants’ social integration patterns and integration strategies, and ensure effective community governance.
